# Proteomic biomarkers in mid-trimester amniotic fluid associated with adverse pregnancy outcomes in patients with systemic lupus erythematosus

**DOI:** 10.1371/journal.pone.0235838

**Published:** 2020-07-17

**Authors:** Hae Sun Jeon, Seung Mi Lee, Young Mi Jung, Sohee Oh, Jin Kyun Park, Eun Bong Lee, Chan-Wook Park, Joong Shin Park, Dohyun Han, Jong Kwan Jun

**Affiliations:** 1 Department of Obstetrics and Gynecology, Seoul National University College of Medicine, Seoul, Korea; 2 Medical Research Collaborating Center, Seoul Metropolitan Government Seoul National University Boramae Medical Center, Seoul, Korea; 3 Division of Rheumatology, Department of Internal Medicine, Seoul National University College of Medicine, Seoul, Korea; 4 Department of Molecular Medicine and Biopharmaceutical Sciences, Graduate School of Convergence Science and Technology, Seoul National University, Seoul, Korea; 5 Proteomics Core Facility, Biomedical Research Institute, Seoul National University Hospital, Seoul, Korea; 6 The Institute of Reproductive Medicine and Population, Medical Research Centre, Seoul National University College of Medicine, Seoul, Korea; University of Mississippi Medical Center, UNITED STATES

## Abstract

We aimed to explore the proteomic profiles of mid-trimester amniotic fluid in pregnant women with systemic lupus erythematosus (SLE) according to the occurrence of adverse pregnancy outcome (APO). The study population included 35 pregnant women with SLE who underwent clinically indicated amniocentesis at 15–24 weeks of gestation. Patients were divided into two groups according to pregnancy outcomes: SLE patients without APO (Group 1) and SLE patients with APO (Group 2). Stored samples of amniotic fluid were analyzed using mass spectrometry (MS)-based proteomics with two-step approach, consisting of discovery and verification phase. In the discovery phase, 44 proteins were differentially expressed between Group 1 and Group 2. In the verification phase, differentially expressed proteins (DEPs) were verified in independent samples using DIA method. Four proteins including filamin A (FLNA), sushi, von Willebrand factor type A, EGF and pentraxin domain containing 1 (SVEP1), lecithin-cholesterol acyltransferase (LCAT), and transglutaminase 2 (TGM2) were differentially expressed both in discovery and verification phase. To select the best combination of proteins for discriminating two groups, three-fold cross validation (CV) with repetition of one hundred times was performed. The multi-marker model with three biomarkers (SVEP1, LCAT, TGM2) had a high discriminatory power to distinguish between the two groups (the area under the receiver operating characteristic, AUROC = 0.946, p <0.001). Our results indicate that the expression of FLNA, SVEP1, LCAT, and TGM2 in mid-trimester amniotic fluid was increased in SLE patients with APO (Group 2). A large-scale prospective study is warranted to verify this finding.

## Introduction

Systemic lupus erythematosus (SLE) is a systemic autoimmune disease with a broad spectrum of symptoms and clinical courses characterized by remissions and flares [1]. It predominantly affects women in their reproductive years, with a female to male incidence ratio of 9:1, peaking at the age of 30–39 years [2, 3]. As SLE has a high prevalence in women of child-bearing age, pregnancy issues are of key interest in clinical practice.

It is well known that pregnant women with SLE are at an increased risk of adverse pregnancy outcomes (APOs), including spontaneous abortion, preeclampsia, intrauterine growth restriction, preterm birth, and fetal death in utero [4–7]. Although the obstetric outcomes have been significantly improved over the last few decades, pregnancy in those people still remains as a high-risk situation [8]. According to the PROMISSE (Predictors of Pregnancy Outcome: Biomarkers in Antiphospholipid Antibody Syndrome and Systemic Lupus Erythematosus) study, the first multicenter, prospective observational study of pregnancies in women with SLE, APOs occurred in 19.0% of pregnancies; fetal death in 4%, neonatal death in 1%, preterm delivery in 9%, and small-for-gestational-age neonate in 10% [9]. Despite the significant impact of SLE on pregnancy outcomes, the mechanisms by which pregnancy complications occur in SLE patients have been complex and incompletely understood.

Amniotic fluid, the innermost space surrounding the fetus, contains a larger amount of fetal- and pregnancy-related proteins than other maternal specimens [10–12]. As a result, amniotic fluid is a rich source of biomarkers, which can give clues on the prediction of APOs for decision making about pregnancy management and delivery planning [13]. Mass spectrometry (MS) based proteomics techniques facilitate uniquely unbiased, sensitive and quantitative analysis of complex biological samples and enable us to better understand the diversity of proteins [14–16].

The main aim of this study, therefore, was to explore possible biomarkers using proteomic analysis of mid-trimester amniotic fluid in pregnant women with SLE. Earlier detection of abnormal pregnancy states will help us predict APOs and properly manage high-risk patients.

## Materials and methods

### Study design and subjects

In this retrospective cohort study, the study population consisted of 35 pregnant women with SLE and met the following criteria: 1) singleton pregnancy; 2) clinically indicated amniocentesis for chromosomal abnormalities at mid-trimester period (15–24 weeks of gestation); 3) stored samples of amniotic fluid available for proteomic profiling; 4) followed up till delivery at Seoul National University Hospital. The study population was divided into two groups according to pregnancy outcomes: 1) SLE patients who had normal pregnancy outcomes (Group 1, SLE patients without APO); 2) SLE patients who had developed APO (Group 2, SLE patients with APO). The Institutional Review Board of Seoul National University Hospital approved this study (No 1611-126-812, date of approval 2017-01-09), and the patients gave their written consent for the collection of data for research purposes.

### Definition of APO

APO was defined by one or more of the following criteria, adopted from PROMISSE study with minor modification [9]: 1) preeclampsia; 2) indicated preterm delivery before 36 weeks of gestation; 3) small for gestational age at birth (<5th percentile) [17]; 4) fetal death in utero, unexplained by chromosomal abnormality, major malformation, or congenital infection; 5) neonatal death before hospital discharge.

### Retrieval of amniotic fluid

After informed consent, amniocentesis was performed under ultrasound guidance. Retrieved amniotic fluid was transferred to laboratories for karyotyping, and the remained amniotic fluid was centrifuged, aliquoted, and stored at -70°C until assayed.

### Proteomic profiling of mid-trimester amniotic fluid

For proteomic analysis of amniotic fluid, we applied in-depth quantitative proteomic strategy, consisting of protein extraction, filter-aided sample preparation, high-pH peptide fractionation based on stage-tip, and high-resolution quadrupole Orbitrap liquid chromatography-tandem mass spectrometry (LC-MS/MS).

**Fig 1:**
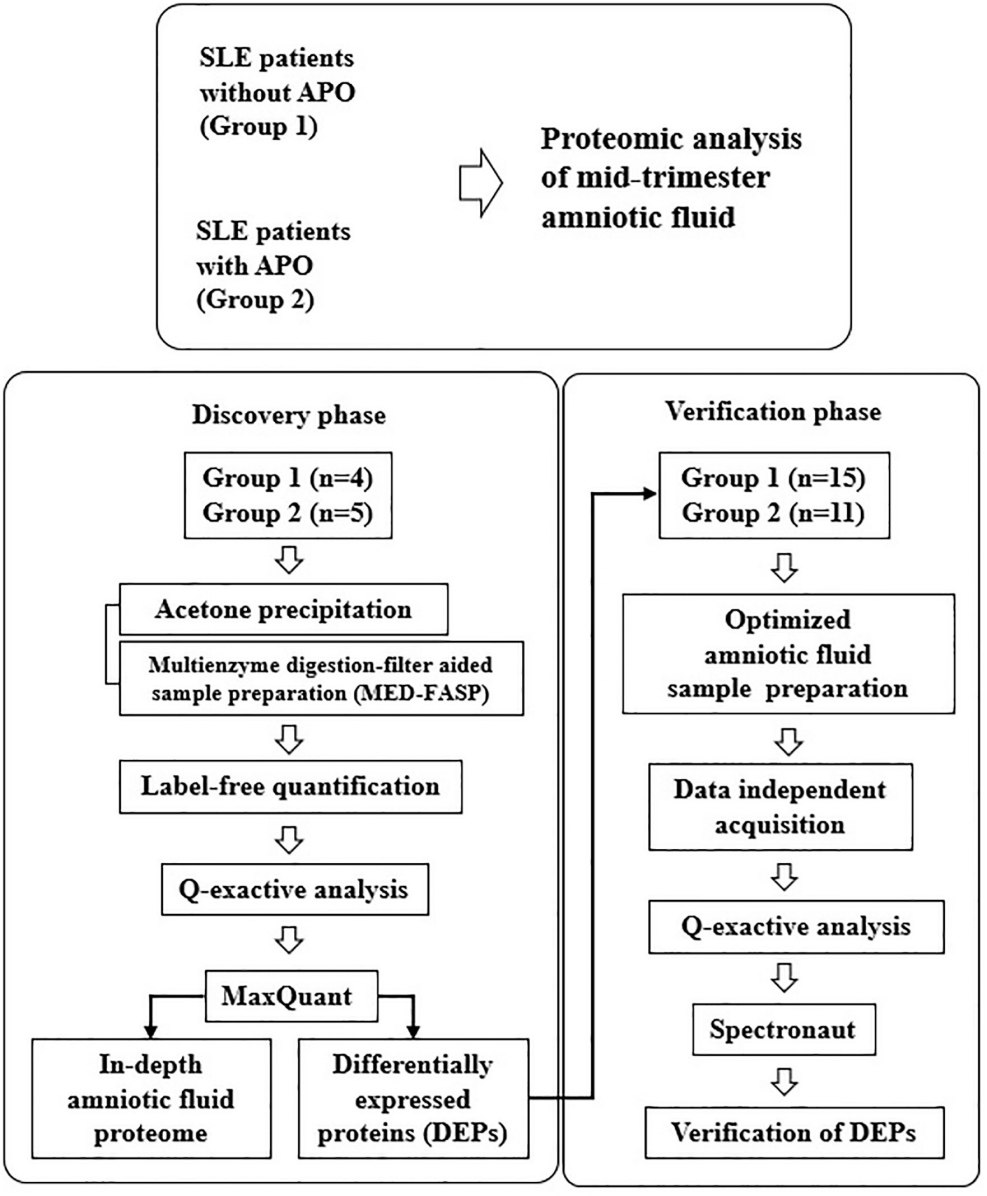
Overall workflow.

For identification of biomarkers in amniotic fluid, we analyzed with two-step approach, consisting of discovery and verification phase (Fig 1). In the discovery phase, we analyzed 9 patients (4 patients in Group 1 and 5 patients in Group 2) with data-dependent acquisition (DDA) approach using Q-exactive Orbitrap mass spectrometry. Label-free quantitative analysis was performed by MaxQuant software, Version 1.6.1.0 [18]. In the verification phase, candidate biomarker targets were verified in independent cohorts consisting of 26 patients (15 patients in Group 1 and 11 patients in Group 2) with data-independent acquisition (DIA) method through hyper reaction monitoring (HRM) peptide kit (Biognosys AG, Switzerland). The data was processed using the Spectronaut software, Version 10 (Biognosys AG, Switzerland) that can be applied to Orbitrap-based mass instruments. Hierarchical clustering was performed based on the Euclidean distance and the average linkage using Perseus software, Version 1.6.0.2 [19]. Detailed explanation of the experimental methods is provided in the S1 File.

### Statistical analysis

Continuous variables were reported as median and range. Categorical variables were presented as the number (%). The differences between two groups were evaluated using the Mann-Whitney U test or Fisher’s exact test as appropriate. Detailed explanation of the statistical analysis of the results of proteomic analysis is provided in the S1 File.

Three-fold cross-validation (CV) was applied to detect the best combination of proteins for discrimination of the two groups and avoid an overfitting problem. In CV, the raw data was randomly partitioned into three subsets to reduce the number of data which can be used for learning the model. After then, two subsets were used as training data for fitting the model, and the remaining subset was assessed as the internal validation data for testing the model. The CV process was repeated three times, with each of subsets used as the validation data. Furthermore, the three-fold CV was repeated 100 times to reduce the possibilities of poor estimates due to chance divisions of the data. For selecting the best combination, all possible models were fit in the training set using logistic regression, and then the predictability of each model was evaluated with the area under the receiver operating characteristic (AUROC) curve. The best combination of proteins was chosen with the highest average-test AUROC. A probability value of <0.05 was considered statistically significant. All statistical analyses were performed using the Statistical Package for the Social Sciences (SPSS) for Windows, Version 25.0 (IBM Corp., Armonk, N.Y., USA) and R for Windows, Version 3.5.1 (http://www.r-project.org).

## Results

### Study population

Among 35 pregnant women with SLE, 16 women (45.7%) developed APO during pregnancy. According to the occurrence of APO, the study population was classified into two groups: 1) SLE patients without APO (Group 1, n = 19); 2) SLE patients with APO (Group 2, n = 16).

Table 1 shows the maternal characteristics. The clinical characteristics including maternal age, parity, body mass index (BMI), and gestational age at amniocentesis were not different between the two groups of cases. However, the SLE patients with APO (Groups 2) had a higher proportion of antiphospholipid syndrome and hypertension than SLE patients without APO (Group 1).

**Table 1:** Characteristics of the study population.

	Group 1 SLE patients without APO (n = 19)	Group 2 SLE patients with APO (n = 16)	P
**Baseline characteristics**			
Maternal age (years)*	34 (27–38)	32 (25–38)	0.301
Nulliparity ^†^	10 (52.6%)	10 (62.5%)	0.734
BMI at sampling (kg/m^2^)*^,^^a^	23.2 (18.3–27.4) (n = 18)	22.9 (17.4–30.8) (n = 13)	0.828
GA at sampling (weeks)*^,^^a^	18.3 (15.7–22.6)	18.3 (16.3–22.0)	0.935
Lupus nephritis ^†^	9 (47.4%)	7 (43.8%)	1.000
Creatinine (mg/dL)*	0.70 (0.45–0.90) (n = 17)	0.75 (0.40–1.60) (n = 14)	0.118
Complement C3 (mg/dL)*	119 (88–171) (n = 15)	94 (32–147) (n = 14)	<0.05
Complement C4 (mg/dL)*	21 (10–33) (n = 15)	10.5 (2–47) (n = 14)	<0.01
Anti-dsDNA (IU/mL) *	6.6 (1.0–58.6) (n = 17)	9.8 (1.0–174) (n = 14)	0.262
Proteinuria ^†^^,^^c^	2/17 (11.8%)	11/14 (78.6%)	<0.001
24-hour urine protein (mg) *	161 (78–510) (n = 4)	2251 (420–18180) (n = 11)	<0.01
Antiphospholipid antibody syndrome ^†^^,^^b^	0 (0%)	6 (37.5%)	<0.01
Presence of lupus anticoagulant ^†^^,^^d^	1 /14 (7.1%)	4/13 (30.8%)	0.165
Hypertension at sampling ^†^^,^^a^^,^^e^	0 (0%)	9 (56.3%)	<0.001

SLE, systemic lupus erythematosus; APO, adverse pregnancy outcome; BMI, body mass index; GA, gestational age

* Values are given as the median (range)

^†^ Values are given as the number (%)

^a^ at sampling: at mid-trimester amniocentesis

^b^ Diagnosis made by attending physician (Rheumatologist or Nephrologist) before mid-trimester amniocentesis

^c^ Proteinuria: ≥1+ on urine protein dipstick or ≥0.3 on random urine protein-creatinine ratio or ≥300 mg on 24-hour urine protein collection

^d^ Positive result before mid-trimester amniocentesis or within 3 months after delivery

^e^ History of hypertension or taking anti-hypertensive medications at mid-trimester amniocentesis

Table 2 summarizes the pregnancy outcomes of the study population. The SLE patients with APO (Group 2) had lower gestational age at delivery and lower birthweight than SLE patients without APO (Group 1). In SLE patients with APO (Group 2), preeclampsia developed in 62.5% of cases, indicated preterm delivery in 68.8%, small for gestational age at birth in 60%, fetal death in utero in 25%, and neonatal death in none of the cases.

**Table 2:** Development of adverse pregnancy outcomes.

	Group 1 SLE patients without APO (n = 19)	Group 2 SLE patients with APO (n = 16)	P
Pregnancy outcomes*			
GA at delivery (weeks)	38.4 (35.7–40.9)	32.6 (20.6–40.3)	<0.001
Birth weight (g)	3000 (2040–3990)	1340 (40–2590)	<0.001
**APO**^†^			
Preeclampsia	0 (0%)	10 (62.5%)	<0.001
Indicated preterm delivery < 36 weeks	0 (0%)	11 (68.8%)	<0.001
Small for gestational age at birth < 5th	0 (0%)	9/15 (60.0%)	<0.001
Fetal death in utero	0 (0%)	4 (25.0%)	<0.05
Neonatal death before discharge	0 (0%)	0 (0%)	(-)

SLE, systemic lupus erythematosus; APO, adverse pregnancy outcome; GA, gestational age

* Values are given as the median (range)

^†^ Values are given as the number (%)

### Global profiling of amniotic fluid in discovery set

**Fig 2:**
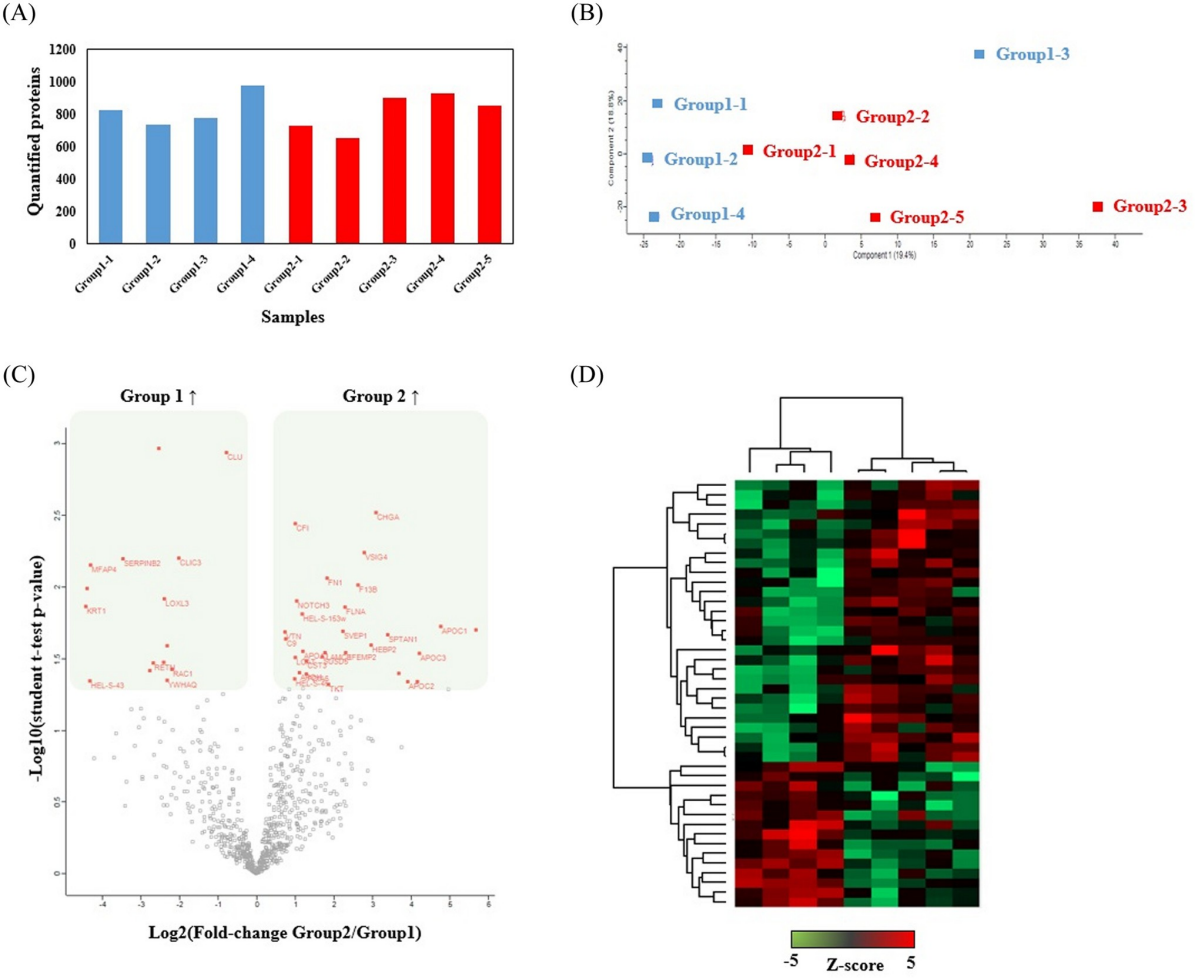
Results of discovery proteomic analysis. (A) Number of quantified proteins in each samples. (B) Principal component analysis (PCA) plot. (C) Volcano plot. (D) Hierarchical clustering.

In the discovery phase analyzing 9 patients (4 patients in Group 1 and 5 patients in Group 2) with DDA approach, a total of 1222 protein groups were identified at protein false discovery rate (FDR) level <1% by in-depth proteomic analysis. Average 800 protein groups were quantified across 9 samples (Fig 2A). Pearson correlation analyses revealed average R values of 0.76 and 0.79 in each group, respectively. Principal component analysis (PCA) was performed using the whole patients’ proteomic expression profile, indicating that cluster of two groups and their corresponding biological replicates were clearly separated (Fig 2B). Label-free quantitation and statistical analysis yielded 44 differentially expressed proteins (DEPs) with p-value <0.05 and fold-change >1.5 (Fig 2C; S1 Table). The proteome profile of 44 protein groups showed a similar separation of two groups (Fig 2D).

### Verification of biomarker candidates using DIA-MS

**Fig 3:**
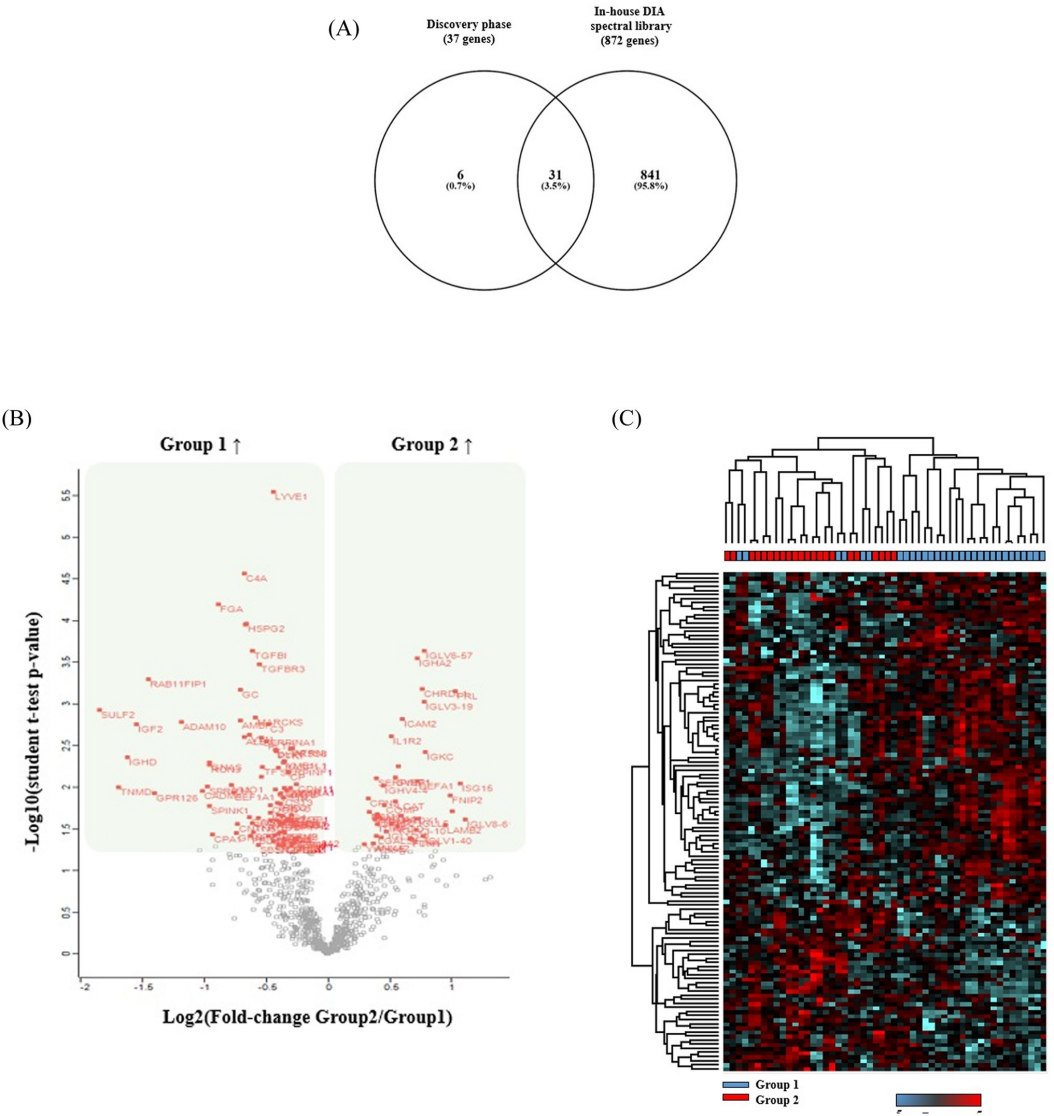
Results of data-independent acquisition (DIA) analysis. (A) Coverage of in-house DIA spectral library. (B) Volcano plot. (C) Hierarchical clustering.

In the verification phase, we analyzed 26 patients (15 patients in Group 1 and 11 patients in Group 2). All 26 samples were analyzed in duplicate with a 90-min LC-DIA/MS method using in-house spectral library. Even though all samples were prepared and analyzed randomly, good reproducibility was observed across LC-DIA/MS runs. On average, we detected 4741 peptides and 815 proteins per sample. Within the DIA data set, 442 proteins were quantified in all 26 samples. Among the 44 biomarker candidates (37 genes), which were developed in the discovery phase, 31 proteins (84%) were detectable in our spectral library (Fig 3A). Statistical analysis of the DIA data showed difference in the composition of the amniotic fluid proteome between the two groups. A pairwise statistical testing based on student t-test yielded 122 DEPs with p-value <0.05 and fold-change >1.5 (Fig 3B; S2 Table). Hierarchical clustering based on 122 proteins that significantly changed clearly separated two sample groups (Fig 3C).

As a result, 10 proteins were identified as overlapped and significantly changed proteins in both discovery and verification phases, and among these, 4 proteins (SVEP1, LCAT, TGM2, and FLNA) showed the same expression pattern (S3 Table).

### Assessment of the discrimination efficacy of amniotic fluid proteins

To select the best combination of proteins for discriminating the two groups, three-fold CV with repetition of 100 times was performed. The multi-marker model with 3 biomarkers (SVEP1, LCAT, TGM2) had a high discriminatory power in distinguishing the two groups (AUROC = 0.946, p <0.001), which was the highest average-test discrimination ability in CV (Table 3).

**Table 3:** Multi-marker models with proteomic biomarkers for discrimination of the two groups.

Variables	Training set			Test set		
	AUC	95% CI		AUC	95% CI	
		Lower	Upper		Lower	Upper
SVEP1	0.676	0.381	0.958	0.680	0.290	0.982
LCAT	0.746	0.502	0.977	0.747	0.405	0.992
FLNA	0.663	0.381	0.942	0.666	0.264	0.979
TGM2	0.758	0.510	0.985	0.757	0.412	0.995
SVEP1+LCAT	0.819	0.616	0.996	0.765	0.438	0.993
SVEP1+FLNA	0.737	0.480	0.979	0.679	0.287	0.983
SVEP1+TGM2	0.896	0.749	1.000	0.847	0.593	0.999
LCAT+FLNA	0.820	0.607	0.998	0.783	0.460	0.996
LCAT+TGM2	0.862	0.683	1.000	0.820	0.541	0.997
FLNA+TGM2	0.826	0.627	0.996	0.756	0.423	0.992
SVEP1+LCAT+FLNA	0.855	0.675	0.999	0.774	0.458	0.989
SVEP1+LCAT+TGM2	0.951	0.863	1.000	0.889	0.706	0.998
SVEP1+FLNA+TGM2	0.925	0.807	1.000	0.831	0.565	0.998
LCAT+FLNA+TGM2	0.907	0.777	1.000	0.823	0.559	0.994
SVEP1+LCAT+FLNA+TGM2	0.971	0.914	1.000	0.843	0.607	0.995

SVEP1, sushi, von Willebrand factor type A, EGF and pentraxin domain containing 1; LCAT, lecithin-cholesterol acyltransferase; FLNA, filamin A; TGM2, transglutaminase 2; AUC, area under the curve; CI, confidence interval

## Discussion

We conducted the present study in pregnant women with SLE to explore possible biomarkers using proteomic analysis in mid-trimester amniotic fluid. The principal findings of this study were as follows: 1) Among 35 pregnant women with SLE, 16 women (45.7%) developed APO; 2) Proteomic analysis of mid-trimester amniotic fluid showed different profiling pattern between the SLE patients without APO (Group 1) and the SLE patients with APO (Group 2); 3) The expression of FLNA, SVEP1, LCAT, and TGM2 was significantly increased in the SLE patients with APO (Group 2) compared with the SLE patients without APO (Group 1).

FLNA is an actin binding protein, which is widely expressed during development [20]. FLNA has been reported to regulate reorganization of the actin cytoskeleton that plays an important role in the formation of several key cellular structures such as the terminal web, microvilli, and cellular junctions, which undergo dynamic changes during early pregnancy [21, 22]. SVEP1 is a large extracellular matrix protein ubiquitously expressed in human tissues and has been characterized as important for cell adhesion [23]. According to a study evaluating the environments of endotoxemia using human umbilical vein endothelial cells, the expression of SVEP1 was significantly upregulated in a human cell culture model of endotoxemia [24]. To date, only one study showed that preeclampsia markers were associated with chorionic villi with low morphology scores and SVEP1 was positively correlated with increasing gestational age during early pregnancy. However, the relationship between preeclampsia and SVEP1 has not been determined [25]. LCAT converts cholesterol and phosphatidylcholines (lecithins) to cholesteryl esters and lysophosphatidylcholines on the surface of high-density lipoproteins (HDLs). LCAT plays an important role in lipoprotein metabolism and circulates in blood plasma as a complex with components of HDL. Cholesterol from peripheral cells is transferred to HDL particles, esterified by the action of LCAT on HDL and then the cholesterol ester is transported to the liver [26]. The plasma lipoprotein profile in the fetus has been demonstrated to be unique in that a relatively larger proportion of cholesterol is carried by HDL particles and HDL metabolism is very important because of the high requirement of the fetus for cholesterol to enable rapid growth [27, 28]. TGM2, a multifunctional enzyme, is implicated in the regulation of cell growth, differentiation, and apoptosis [29]. TGM2 has the capability both to facilitate and to prevent apoptosis, and these two opposing activities occur distinctly depending on the specific biochemical pathways [30–32].

Although the mechanisms underlying obstetric complications in SLE patients are poorly understood, evidence suggests that the thrombotic damage to the utero-placental vasculature plays a central role in the pathogenesis of the deterioration of fetal wellbeing [33]. Others hypothesized to explain the occurrence of the APO by adversely affecting trophoblast functions, impairing this invasion, differentiation, and maturation [34]. A poor placentation has been demonstrated to impair the physiologic spiral arteries remodeling and their change into low-resistance vessels during the first half of pregnancy [35]. Further mechanisms such as direct cellular injury, apoptosis and inhibition of proliferation and syncytia formation involved in defective placentation [36]. Moreover, oxidative stress by cross-reaction with oxidized LDL and complement activation can be considered the contributing factors in placental injury, suggesting an autoimmune inflammatory role in obstetric complications [37]. The increased amniotic fluid proteins found in the current study (FLNA, SVEP1, LCAT, and TGM2) may be the key components constituting these pathogenic mechanisms, but further studies are needed to demonstrate the exact mechanism of these components in the development of APO in SLE patients.

To our best knowledge, this is the first study conducting proteomic analysis of amniotic fluid in pregnant women with SLE. A number of proteomic studies have been performed in non-pregnant patients with SLE. SLE is a systemic autoimmune disease, which affects almost all organs and tissues, and a variety of sample types, such as serum, urine, skin biopsy, and cerebrospinal fluid, obtained from SLE patients was used. However, there is a paucity of information regarding the potential proteomic biomarkers in amniotic fluid of SLE patients during asymptomatic mid-trimester period. As the composition of amniotic fluid is modified throughout pregnancy, its protein profile reflects the physiological and pathological changes that affect both the mother and the fetus. It may provide valuable information on developing fetus and intrauterine environment and be a rich source of biomarkers for earlier detection of abnormal pregnancy states. Proteins are indeed important executors of physiological functions, and variations in their expression reflect the different conditions. Therefore, proteomic analysis of amniotic fluid is an important tool to select cases complicated by APO, which require different treatment approaches. To this end, we investigated the differently expressed proteins in the amniotic fluid of SLE patients according to their obstetric outcomes using LC-MS/MS, although further studies are needed to confirm our experimental data. There is an urgent need to improve our knowledge about identifying the potential predictors reflecting abnormal pregnancy state in pregnant women with SLE, allowing for earlier intervention to improve perinatal outcome. Our results may contribute to the future development of biomarkers to reliably estimate the subsequent development of APOs.

One of the limitations of our study was the limited volume of amniotic fluid samples. In the current study, it made verification of multiple biomarkers by western blot or enzyme-linked immunosorbent assay (ELISA) problematic. To overcome this issue, we designed two step approaches in proteomic analysis, consisting of discovery and verification phase. In the discovery phase, we analyzed 9 patients for screening for possible proteomic biomarkers with DDA approach. In the verification phase, we analyzed in independent 26 patients (15 patients in Group 1 and 11 patients in Group 2) and confirmed that 4 proteins (SVEP1, LCAT, TGM2, and FLNA) were also significantly different in verification set. In other previous studies, targeted MS approaches allowed quantifying tens of protein candidates simultaneously using small amount of proteins (for example, ng to μg) [38, 39]. Recently, DIA, one of the targeted MS approach, has emerged as a powerful quantification methodology with improved sensitivity and reproducibility [40]. Therefore, we performed DIA approach to verify the discovered candidate biomarkers in amniotic fluid samples of 26 patients. Other limitations include lack of validation of our experimental data in an independent cohort. Further validation of these potential biomarkers in pregnant women with SLE on large independent cohorts would be required to develop reproducible and reliable protein biomarkers that may discriminate the presence or absence of abnormal pregnancy states.

## Conclusions

In summary, we performed proteomic analysis to investigate the possible proteomic biomarkers in mid-trimester amniotic fluid of SLE patients using LC-MS/MS analyses. As a result, four proteins including FLNA, SVEP1, LCAT, and TGM2 were differentially expressed proteins in SLE patients with APO and may be used for further investigations. And a multi-marker model with three biomarkers (SVEP1, LCAT, TGM2) had a high discriminatory power to distinguish between the two groups. Further studies are required to confirm these observations, determine the accurate role of these proteins in the pathogenesis of APO in SLE patients, and develop reliable predictors for estimating subsequently APO.

## Supporting information

S1 Table List of the differentially expressed proteins (DEPs) in discovery phase. (XLSX)

S2 Table List of the differentially expressed proteins (DEPs) in verification phase. (XLSX)

S3 Table List of the differentially expressed proteins (DEPs) overlapped in both discovery and verification phases. (XLSX)

S1 Data Demographic characteristics and pregnancy outcomes of the study population. (SAV)

S1 File Supplementary file. (DOCX)
