# Supplementary material for: Proteomic biomarkers in mid-trimester amniotic fluid associated with adverse pregnancy outcomes in patients with systemic lupus erythematosus
Source: PLoS One. 2020 Jul 17;15(7):e0235838. doi: 10.1371/journal.pone.0235838 (PMC7367458; doi:10.1371/journal.pone.0235838)
Supplement: S1 File — (DOCX) [file pone.0235838.s005.docx]

**Supplementary file**

**Amniotic fluids sample preparation**

After centrifugation of amniotic fluid at 15,000 rpm for 10min at 4℃, the protein content of supernatants was measured by bicinchoninic acid (BCA) assay. For label-free quantification in the discovery phase, 300 ug of amniotic fluid proteins were precipitated by adding a 6-fold volume of ice-cold acetone prior to the digestion step. For data-independent acquisition (DIA) analysis in the verification phase, 200 ug of amniotic fluid proteins per individual sample were used. Protein digestion was performed via the 2-step Filter Aided Sample Preparation (FASP) procedure as described with some modifications.[[1](#_ENREF_1), [2](#_ENREF_2)] Protein pellets were resolved in SDT buffer (2% SDS, 10mM TCEP, and 50mM CAA in 0.1M Tris pH 8.0) and loaded onto a 10K Amicon filter (Milipore). The buffer was exchanged with UA solution (8M urea in 0.1M Tris pH 8.5) via centrifugation at 14,000 x g. Following the exchange of buffer with 40mM ammonium bicarbonate (ABC), protein digestion was performed at 37℃ overnight using a trypsin/LysC mixture at a 100:1 protein-to-protease ratio. The digested peptides were collected by centrifugation. After the filter units were washed with 40 mM ABC, second digestion was performed at 37℃ for 2 hours using trypsin (enzyme-to-substrate ratio [w/w] of 1:1000). All resulting peptides were acidified with 10% Trifluoroacetic acid (TFA) and desalted using homemade C18-StageTips as described.[[1](#_ENREF_1), [2](#_ENREF_2)] Desalted samples were completely dried with a vacuum dryer and stored at -80℃.

**High-pH StageTip-based peptide fractionation**

**For label-free quantification in discovery phase and peptide spectrum library in verification phase, StageTip-based, high-pH peptide fractionation was performed as described with some modifications.[**[**1**](#_ENREF_1)**] Peptides obtained from pooled samples were dissolved in 200 ul of loading solution (10 mM ammonium hydroxide solution, pH 10 and 2% acetonitrile) and separated on the reversed-phase tip columns, prepared by packing POROS 20 R2 (Invitrogen, Carlsbad, CA) into a 200-ul yellow tip with C18 Empore disk membranes (3M, Bracknell, UK) at the bottom. After conditioning of microcolumns with methanol, acetonitrile, and loading buffer, peptides were loaded at pH 10, and 20 fractions were subsequently eluted with buffer solutions, pH 10, containing 5%, 10% 15%, 20%, 25%, 30%, 35%, 40%, 60%, and 80% acetonitrile. To improve the orthogonal fractionation of the reverse phase (RP) – reverse phase (RP) separation, 20 fractions were combined into six fractions in a noncontiguous manner. The six fractions were dried in a vacuum centrifuge and stored at -80℃ until LC-MS/MS analysis.**

**LC-MS/MS analysis**

**All LC-MS/MS analysis including DDA and DIA methods was performed using Quadrupole Orbitrap mass spectrometers, Q-exactive plus (Thermo Fisher Scientific, Waltham, MA) coupled to an Ultimate 3000 RSLC systems (Dionex) via a nano electrospray source, as described with some modifications.[**[**1**](#_ENREF_1)**,** [**3**](#_ENREF_3)**] Peptide samples were separated on the 2-column setup with a trap column (75 um I.D. x 2 cm, C18 3 um, 100 Å) and an analytical column (50 um I.D. x 15 cm, C18 1.9 um, 100 Å). Prior to sample injection, the dried peptide samples were redissolved in solvent A (2% acetonitrile and 0.1% formic acid). After the samples were loaded onto the nano LC, a 180-minute gradient from 8% to 26% solvent B (100% acetonitrile and 0.1% formic acid) was applied to all samples. The spray voltage was 2.0 kV in the positive ion mode, and the temperature of the heated capillary was set to 320°C. Mass spectra were acquired in data-dependent mode using a top 15 method on a Q Exactive. The Orbitrap analyzer scanned precursor ions with a mass range of 300–1650 m/z and a resolution of 70,000 at m/z 200. Higher-energy collisional dissociation (HCD) scans were acquired on the Q Exactive at a resolution of 17,500. HCD peptide fragments were acquired at a normalized collision energy (NCE) of 27. The maximum ion injection time for the survey and MS/MS scans was 20 ms and 120 ms, respectively.**

**The DIA method consisted of a survey scan at 35,000 resolution from 400 to 1,220 m/z (AGC target of 3x106 or 60ms injection time). Then, 19 DIA windows were acquired at 35,000 resolutions with automatic gain control target 3e6 and auto for injection time).[**[**4**](#_ENREF_4)**] Stepped collision energy was 10 % at 27%. The workflow of LC-MS/MS for identification and quantification of proteins in amniotic fluids is depicted in Figure 1.**

**Data processing for Label-free quantification**

**Mass spectra were processed in MaxQuant (version 1.5.3.1).[**[**5**](#_ENREF_5)**] MS/MS spectra were searched against Uniprot Human Database (December 2014, 88,657 entries) using the Andromeda search engine.[**[**6**](#_ENREF_6)**] Primary searches were performed using a 6-ppm precursor ion tolerance for total protein level analysis. The MS/MS ion tolerance was set to 20 ppm. Cysteine carbamido-methylation was set as a fixed modification. N-acetylation of protein and oxidation of methionine were set as variable modifications. Enzyme specificity was set to full tryptic digestion. Peptides with a minimum length of six amino-acids and up to two missed-cleavages were considered. The required false discovery rate (FDR) was set to 1% at the peptide, protein, and modification level. To maximize the number of quantification events across samples, we enabled the ‘Match between Runs’ option on the MaxQuant platform.**

**Data processing for the DIA MS**

**For generation of the spectral libraries, 18 DDA measurements of the pooled sample groups were performed. DDA spectra were searched using the Maxquant against Uniprot Human Database (December 2014, 88,657 entries) and the iRT standard peptides sequence. A spectral library was generated using spectral library generation in Spectronaut 10. The DIA data of individual samples were analyzed with Spectronaut 10 (Biognosys, Schlieren, Switzerland). First, we converted the DIA raw files into an htrm format by using the GTRMS Converter provided by the Spectranaut. The FDR was estimated with the mProphet approach and set to 1% at peptide precursor level and at 1% at protein level.[**[**7**](#_ENREF_7)**] The proteins were inferred by the software, and the quantification information was acquired at the protein level by using the q-value < 0.01 criteria, which was used for subsequent analyses.**

**Statistical analysis for DEP selection**

**For quantitative analysis in DDA data, we first filtered out proteins with at least 70% quantified values in each group. Missing values were imputed on the basis of a normal distribution (width = 0.3, down-shift = 1.8) to simulate signals of low abundance proteins. Finally, data were normalized using width adjustment, which subtracts the medians and scales all values in a sample to have equal interquartile ranges.[**[**8**](#_ENREF_8)**] Two-sided t-tests were performed with p-value cut-off of 0.05 and fold-change > 1.5 to identify the differentially expressed proteins (DEPs) between Group1 and Group2. The normalized protein abundances were subjected to z-normalization followed by hierarchical clustering with pearson correlation distance. DIA data was also processed as described above. All statistical analyses in DDA and DIA data were performed using Perseus software.[**[**9**](#_ENREF_9)**]**

**References**

1. Han D, Jin J, Woo J, Min H, Kim Y. Proteomic analysis of mouse astrocytes and their secretome by a combination of FASP and StageTip-based, high pH, reversed-phase fractionation. Proteomics. 2014;14(13-14):1604-9.

2. Woo J, Han D, Park J, Kim SJ, Kim Y. In-depth characterization of the secretome of mouse CNS cell lines by LC-MS/MS without prefractionation. Proteomics. 2015;15(21):3617-22.

3. Lee H, Kim K, Woo J, Park J, Kim H, Lee KE, et al. Quantitative Proteomic Analysis Identifies AHNAK (Neuroblast Differentiation-associated Protein AHNAK) as a Novel Candidate Biomarker for Bladder Urothelial Carcinoma Diagnosis by Liquid-based Cytology. Molecular & cellular proteomics : MCP. 2018;17(9):1788-802.

4. Bruderer R, Bernhardt OM, Gandhi T, Miladinovic SM, Cheng LY, Messner S, et al. Extending the limits of quantitative proteome profiling with data-independent acquisition and application to acetaminophen-treated three-dimensional liver microtissues. Molecular & cellular proteomics : MCP. 2015;14(5):1400-10.

5. Tyanova S, Temu T, Cox J. The MaxQuant computational platform for mass spectrometry-based shotgun proteomics. Nature protocols. 2016;11(12):2301-19.

6. Cox J, Neuhauser N, Michalski A, Scheltema RA, Olsen JV, Mann M. Andromeda: a peptide search engine integrated into the MaxQuant environment. Journal of proteome research. 2011;10(4):1794-805.

7. Reiter L, Rinner O, Picotti P, Huttenhain R, Beck M, Brusniak MY, et al. mProphet: automated data processing and statistical validation for large-scale SRM experiments. Nature methods. 2011;8(5):430-5.

8. Deeb SJ, Tyanova S, Hummel M, Schmidt-Supprian M, Cox J, Mann M. Machine Learning-based Classification of Diffuse Large B-cell Lymphoma Patients by Their Protein Expression Profiles. Molecular & cellular proteomics : MCP. 2015;14(11):2947-60.

9. Tyanova S, Temu T, Sinitcyn P, Carlson A, Hein MY, Geiger T, et al. The Perseus computational platform for comprehensive analysis of (prote)omics data. Nature methods. 2016;13(9):731-40.
